# Synergistic Effect and Mechanism of Combined Astaxanthin and Curcumin Administration on Ovarian Function in PCOS Mice

**DOI:** 10.1002/fsn3.4597

**Published:** 2024-11-20

**Authors:** Gao Zhang, Man He, Zun Wang, Jinrong Zheng, Dan Zhao, Li Nie, Dongzhi Yuan, Ziyu Zhao, Wei Wang, Limin Yue

**Affiliations:** ^1^ Department of Physiology, West China School of Basic Medical Sciences and Forensic Medicine Sichuan University Chengdu China; ^2^ South China Biopharmaceutical (Shenzhen) Co, Ltd Shenzhen China; ^3^ Reproductive Endocrinology and Regulation Joint Laboratory, West China Second Hospital Sichuan University Chengdu China

**Keywords:** astaxanthin, curcumin, ovarian function, polycystic ovary syndrome

## Abstract

To investigate the synergistic effect of astaxanthin and curcumin on ovarian function in polycystic ovary syndrome (PCOS) mice and to elucidate the underlying mechanisms, fifty 4‐week‐old female mice were randomly divided into five groups: (i) normal control group; (ii) PCOS model group; (iii) PCOS + astaxanthin group; (iv) PCOS + curcumin group; and (v) PCOS + astaxanthin–curcumin. Throughout the study, various parameters were meticulously evaluated, including serum levels of key reproductive hormones (testosterone (T), estradiol (E2), follicle‐stimulating hormone (FSH), luteinizing hormone (LH), and anti‐Müllerian hormone (AMH)), as well as monitoring alterations in the estrous cycle, follicle development, and ovulation rates. Additionally, markers of oxidative stress and inflammation were measured. Findings revealed that PCOS mice exhibited disordered estrous cycle, polycystic ovarian morphologies, abnormal serum reproductive hormones and lipid metabolism, heightened oxidative stress, and augmented inflammatory responses. Notably, upon administration of the combined astaxanthin and curcumin, PCOS mice exhibited significant improvements in their estrous cyclicity and ovarian histology. The treatment led to a significant reduction in serum total cholesterol (TC) levels and normalization of T, E2, FSH, LH, and AMH levels. Moreover, there was a marked decrease in the levels of serum reactive oxygen species (ROS) and malondialdehyde (MDA), indicating attenuation of oxidative stress. Concurrently, the activity of the antioxidant enzyme catalase (CAT) significantly increased, while proinflammatory cytokines interferon‐γ (IFN‐γ) and interleukin‐6 (IL‐6) in the ovarian tissue were notably decreased. The combined treatment also resulted in a substantial increase in the number of ova and a significant decline in the rate of abnormal ova, with these therapeutic effects proving more effective compared to either astaxanthin or curcumin alone. In conclusion, the co‐administration of astaxanthin and curcumin demonstrated a remarkable effect in improving abnormal lipid metabolism and restoring reproductive endocrine functions in PCOS mice. Furthermore, it effectively mitigated systemic and local oxidative stress and chronic inflammatory injury, thereby promoting follicular growth and enhancing ovulatory function in the context of PCOS.

## Graphical abstract

In conclusion, the administration of astaxanthin and curcumin demonstrated a remarkable capacity to ameliorate the aberrant lipid metabolism and restore reproductive endocrine functions in PCOS mice. Furthermore, it effectively mitigated systemic and local oxidative stress and chronic inflammatory injury, thereby promoting follicular growth and enhancing ovulatory function in the context of PCOS.

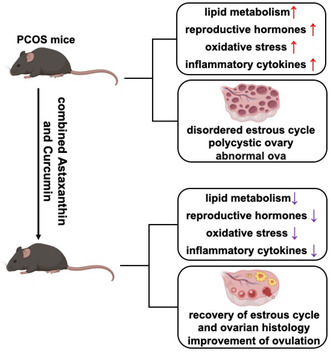

Polycystic ovary syndrome (PCOS) is one of the most prevalent reproductive endocrine disorders affecting women during their reproductive years, with an estimated prevalence reaching up to 5%–10% among this demographic (Conway et al. 2014). This condition exhibits a highly diverse array of clinical presentations, characterized predominantly by hyperandrogenemia, ovarian polycystic morphological changes, and irregular or absent ovulation patterns (Lim et al. 2013). The impact of PCOS extends beyond reproductive impairment, significantly affecting the physical and psychological well‐being of affected women (Lim et al. 2013). Therefore, identifying dietary supplements that can improve ovarian function in individuals with PCOS is of great significance.

Oxidative stress injury and chronic low‐grade inflammation are implicated in the etiology of numerous diseases and exert considerable influence over the pathological procession of PCOS (Mohammadi 2019; Chen and Zhong 2014; Senoner and Dichtl 2019). Prior research has demonstrated that within the ovarian follicular growth microenvironment, an intricate balance exists between oxidative and antioxidative systems (An et al. 2021). Specifically, an increase in reactive oxygen species (ROS) levels within ovarian tissues, coupled with a decline in the antioxidant capacity of oocytes, inevitably leads to oxidative stress damage. This damage disrupts follicular integrity, impairs follicle maturation, and inhibits ovulation, which are important pathological characteristics of PCOS. Indeed, clinical evidence demonstrates that PCOS patients exhibit elevated serum markers of oxidative stress relative to healthy individuals (Macut, Bjekić‐Macut, and Savić‐Radojević 2013). Our research team has previously highlighted that excessive oxidative stress resulting from the hyperactivity of the polyol pathway in PCOS mice negatively impacts the follicular developmental microenvironment, culminating in impaired follicle growth and disrupted ovulation. This disruption constitutes a pivotal factor contributing to the follicular developmental abnormalities observed in PCOS mice. Notably, inhibiting aldose reductase (ADR), the key enzyme of the polyol pathway, effectively diminishes oxidative stress reactions and significantly enhances ovarian function (Wang et al. 2023). Hence, it is evident that oxidative stress represents a central component in the pathogenesis of PCOS.

Since Kelly initially revealed in 2001 that PCOS patients exhibit a certain extent of chronic inflammation, the significance of inflammation theory in the pathogenesis of PCOS has gradually attracted people's attention and become a hot spot in PCOS research. Comparative studies have demonstrated that, compared to healthy women, those with PCOS patients significantly elevated circulating levels of inflammatory biomarkers (Abraham Gnanadass, Divakar Prabhu, and Valsala Gopalakrishnan 2021). Notably, several findings indicate that hyperandrogenemia characteristic of PCOS might be directly instigated by inflammation, establishing a strong correlation between hyperandrogenism and chronic low‐grade inflammation (González 2012). This underscores the pivotal role that persistent, low‐level inflammation plays in the pathophysiology of PCOS. As a result, the exploration and utilization of dietary resources containing inherent antioxidant properties as therapeutic interventions for such conditions have become critically important.

In recent years, the effect of astaxanthin on the reproductive system has increasingly come under the spotlight of the scientific community. Its unique molecular configuration confers an antioxidant potency that surpasses that of numerous other naturally occurring antioxidants. Feeding regimens fortified with astaxanthin have demonstrated remarkable efficacy in enhancing the antioxidant defense within chicken ovaries, elevating serum concentrations of reproductive hormone, augmenting follicle numbers, and concomitantly reducing the prevalence of atresia in secondary follicles (Pertiwi, Nur Mahendra, and Kamaludeen 2022).

Curcumin (CUR), a polyphenolic compound derived from the traditional Chinese medicinal herb turmeric, exhibits a multitude of pharmacological actions, including potent anti‐inflammatory, antitumorigenic, antiatherosclerotic, antioxidant, and regulatory effects on lipid metabolism. Studies have indicated that CUR exerts a restorative influence on ovarian insufficiency and confers a protective benefit to the ovaries (Do et al. 2015).

In this study, PCOS model mice were used to examine the synergistic effects and underlying mechanisms of astaxanthin combined with CUR on ameliorating ovarian dysfunction in PCOS mice, providing experimental basis for clinical PCOS adjuvant therapy.

## 1 Materials and Methods

### 1.1 Experimental Animals

Four‐week‐old female C57/BL/6 mice were purchased from Chengdu Dashuo Biotechnology Co. Ltd. (SCXK (Sichuan) 2020–030). These mice were maintained under standardized environmental conditions, characterized by a constant temperature of 20°C and a 12‐h light/dark cycle, with unrestricted access to food and water. The entire study protocol received ethical approval from the Institutional Animal Care and Use Committee (IACUC) of Sichuan University. Throughout the experiment, every feasible measure was taken to alleviate and minimize any potential suffering experienced by the animals involved in the study. Cervical dislocation is a commonly used method of mouse euthanasia. In this study, all mice were eventually sacrificed by cervical dislocation to minimize their suffering as much as possible.

### 1.2 The Main Reagents

Astaxanthin and CUR were provided by FARMNAN Biopharmaceutical (Shenzhen) Co. Ltd. The 60% high‐fat diet, designated as H10060, was purchased from Beijing Huafukang Biotechnology Co. Ltd. (SCXK (Beijing) 2014–0008). PMSG and HCG were purchased from Chifeng Born Pharmaceutical Company. 4% paraformaldehyde (BL539A), eosin, and hematoxylin (BL700A) were purchased from Biosharp. Hyaluronidase (G1115000) was purchased from Sigma‐Aldrich. The mouse interferon γ (IFN‐γ) ELISA detection kit (CB10139‐Mu) was purchased from Shanghai Keaibo Biotech, while the mouse interleukin 6 (IL‐6) ELISA kit (ml098430) was purchased from MLBio. The mouse ROS ELISA detection kit (CB10366‐Mu), mouse MDA ELISA detection kit (CB10205‐Mu), and mouse superoxide dismutase (SOD) ELISA detection kit (CB10221‐Mu) were purchased from Shanghai Keaibo Biosciences. The mouse catalase (CAT) ELISA kit (ml037752) was also purchased from MLBio. Dihydroethidium (a superoxide anion fluorescent probe, S0063) was purchased from Beyotime Biotechnology.

### 1.3 Experimental Design and Animal Grouping

Fifty 4‐week‐old female mice were systematically randomized into five groups, each consisting of 10 mice: (i) normal control group; (ii) PCOS model group (DHEA combined with high‐fat diet); (iii) astaxanthin group alone (PCOS mice received 1.6 mg/piece/day astaxanthin); (iv) CUR group alone (PCOS mice received 0.1 mg/piece/day CUR); and (v) astaxanthin–CUR combination group (combo, PCOS mice received 1.6 mg/day astaxanthin + 0.1 mg/day and CUR). Following a 21‐day period of modeling induction, a series of assessments were performed. These included measuring the serum levels of reproductive hormones (testosterone [T], estradiol [E2], follicle‐stimulating hormone [FSH], luteinizing hormone [LH], and anti‐Müllerian hormone [AMH]), lipid metabolism indexes (total cholesterol [TC], triglyceride [TG], high‐density lipoprotein cholesterol [HDL‐C], and low‐density lipoprotein cholesterol [LDL‐C]), alterations in the estrous cycle, ovarian follicular development, and ovulation rates. Moreover, the study also evaluated oxidative stress indices in both serum and ovaries (ROS, MDA, SOD, and CAT) along with ovarian expression levels of inflammatory cytokines (IFN‐γ and IL‐6).

### 1.4 Determination of Vaginal Smear and Estrous Cycle in Mice

Following the gentle injection of normal saline into the mouse's vagina and subsequent aspiration in triplicate, the collected fluid is carefully dispersed onto a microscope slide, thereby enabling the microscope examination of the composition of exfoliated vaginal cells. In mice, the normal estrous cycles span approximately 4–5 days and are divided into dioestrus, proestrus, estrous, and postestrous phases. The characteristics of vaginal smears in each period are as follows: During the dioestrus phase, the vaginal smears predominantly display a substantial presence of leukocytes, reflecting a high white blood cell count. In the proestrus stage, the smears are characterized by a preponderance of irregularly shaped nucleated epithelial cells, accompanied by a smaller quantity of leukocytes and keratinized, nonnucleated epithelial cells. During estrous, the samples are marked by a majority of keratinized, nonnucleated epithelial cells, which are indicative of this particular phase. Finally, in the postestrous phase, the smears once again show a significant number of leukocytes, coupled with the presence of keratinized, nonnucleated epithelial cells.

### 1.5 Mouse Ovulation Induction Experiment

Female mice were administered intraperitoneal injections of 10 IU of pregnant mare serum gonadotropin (PMSG), followed by a subsequent intraperitoneal injection of another 10 IU of human chorionic gonadotropin (hCG) precisely 48 h later. 16 h post‐hCG administration, the mice were euthanized, and their oviducts were meticulously excised and placed into Petri dishes filled with M2 buffer solution. To liberate the oocyte–cumulus complex, the oviducal ampulla was gently perforated using a fine needle. Subsequently, the cumulus cells surrounding the oocytes were enzymatically dissolved by treating the complexes with 0.2% hyaluronidase. Upon removal of the cumulus layer, all oocytes were transferred into fresh Petri dishes for the purpose of quantifying the number of oocytes and examining their morphological integrity.

### 1.6 HE Staining of the Mouse Ovaries

Ovarian tissues from mice were harvested, fixed in 4% paraformaldehyde, embedded in paraffin, sectioned, and subjected to routine dehydration. The sections were subsequently stained with hematoxylin and eosin, and the slides were microscopically evaluated following mounting.

### 1.7 Detection of Reproductive Hormones in Mouse Serum

In this investigation, radioimmunoassay techniques were employed to quantify the reproductive hormones FSH, LH, T, E2, P4, and AMH in the serum of mice.

### 1.8 Measurement of Serum Lipid Metabolism Indices in Mice

Biochemical assays were used in this work to determine the concentrations of TC, TG, HDL‐C, and LDL‐C in the serum of mice.

### 1.9 Assessment of Oxidative Stress in Mouse Serum

This study utilized ELISA kits to measure the levels of ROS, SOD, CAT, MDA, and overall serum oxidative stress status in mice.

### 1.10 Detection of Oxidative Stress Markers in Mouse Ovarian Tissue

Here, the levels of the oxidative stress indicators SOD, CAT, and MDA in mouse ovarian tissues were quantified using ELISA kits specifically designed for tissue samples.

### 1.11 Fluorescent Staining of ROS in Mouse Ovarian Tissue Stained With Dihydroethidium

For ROS visualization, ovarian tissue sections from mice were incubated with dihydroethidium (a superoxide anion fluorescent probe) at 37°C for 30 min. Following proper washing, the tissues were inspected under a fluorescence microscope.

### 1.12 Determination of Inflammatory Factors in Mouse Ovaries

The levels of the inflammatory factors IFN‐γ and IL‐6 in mouse ovarian tissues were assayed using ELISA kits in this study.

### 1.13 Statistical Analysis

All experimental data were expressed as the mean ± SD (Mean ± SD). Statistical processing was conducted using GraphPad Prism (version 5.0). Data homogeneity of variance was tested first, and then for comparison between two groups, the *t*‐test was applied. For evaluations among three or more groups, following a one‐way ANOVA test, Tukey's HSD test was used. The threshold for statistical significance was set at 0.05, where *p* values < 0.05 were deemed statistically significant.

## 2 Results

### 2.1 The Synergistic Use of Astaxanthin and CUR Effectively Rectifies Estrous Cycle Irregularities in PCOS Mice

**FIGURE 1:**
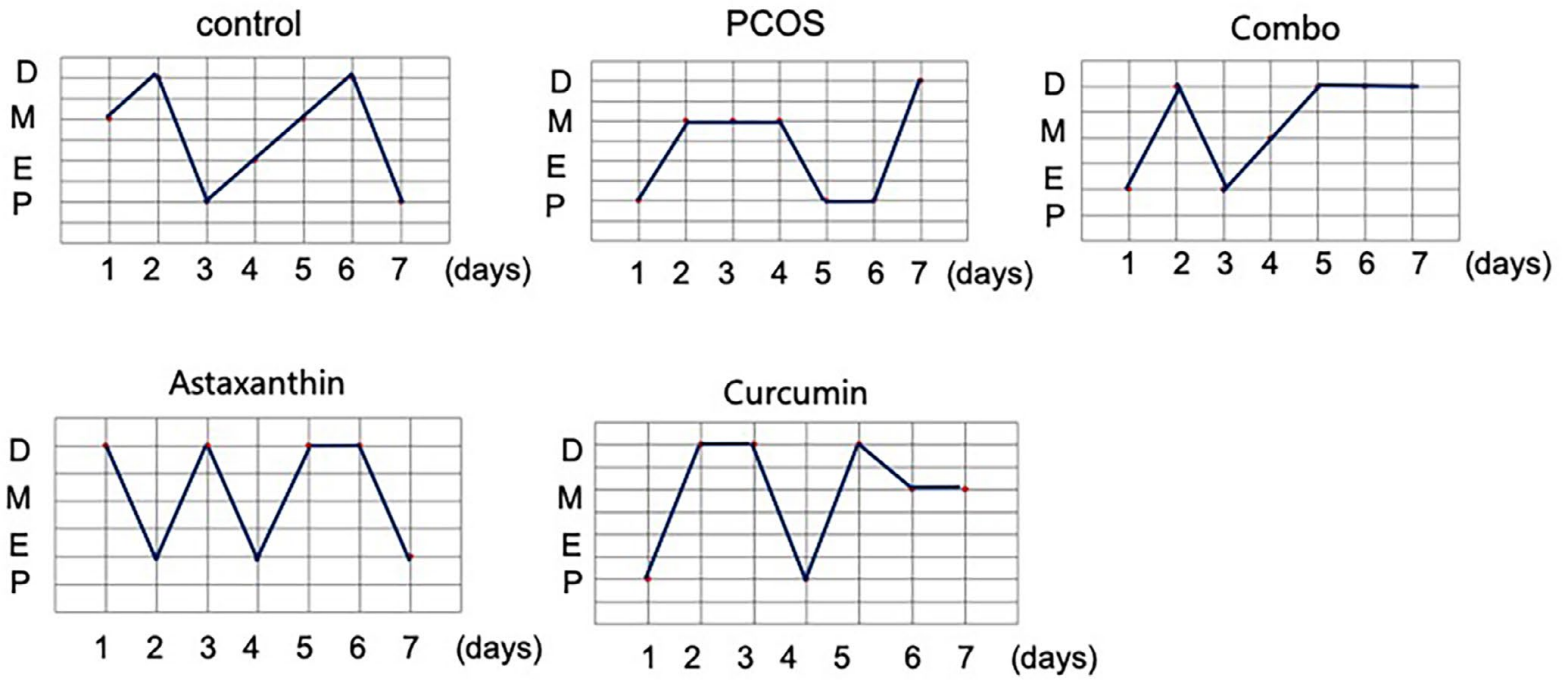
The estrous cycle in mice. The estrous cycle in mice of control, PCOS, combo, astaxanthin, and curcumin groups. Combo, astaxanthin combined with curcumin; PCOS, Polycystic ovary syndrome.

When juxtaposed against the normal control group, PCOS mice exhibited conspicuous disturbances in their estrous cycles. However, upon comparing the PCOS group with the combination therapy group (referred to as combo), it was observed that the latter showed a significantly enhanced estrous cycle pattern, with improvements that were notably more pronounced than those observed in mice treated solely with either astaxanthin or CUR alone (Figure 1).

These results indicate that the coadministration of astaxanthin and CUR significantly ameliorates estrous cycle abnormalities in PCOS mice, and achieves a superior outcome compared to when either compound is administered individually.

### 2.2 The Synergistic Use of Astaxanthin and CUR Significantly Improve Ovarian Morphology in PCOS Mice

**FIGURE 2:**
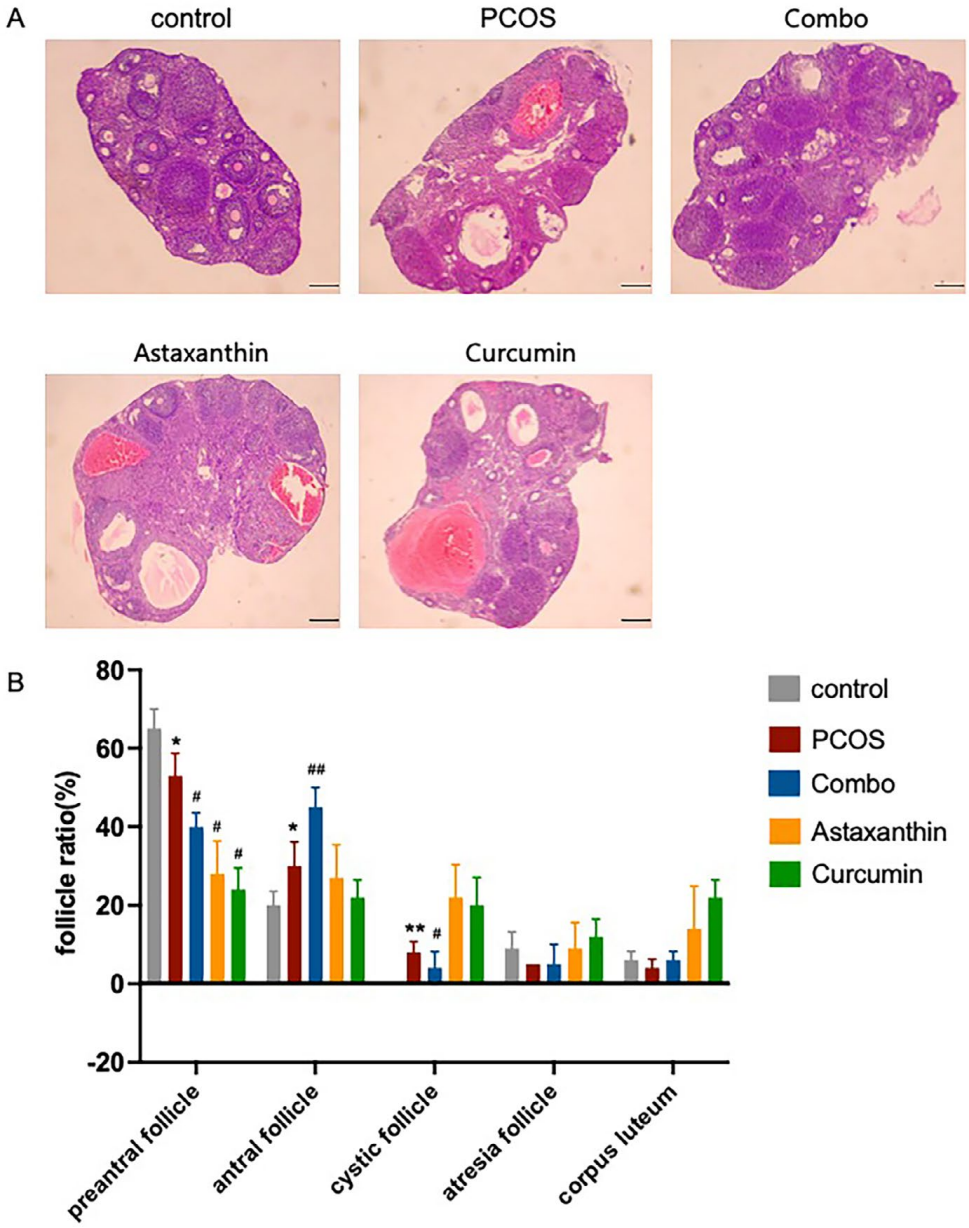
HE staining of mouse ovaries and statistical analysis. (A) HE staining of mouse ovaries of control, PCOS, combo, astaxanthin, and curcumin groups; (B) statistical analysis of Figure 2A. Combo, astaxanthin combined with curcumin; PCOS, Polycystic ovary syndrome.

Relative to the normal control group, the PCOS mice displayed evident polycystic ovarian changes. Nevertheless, when contrasted with the PCOS group, the mice receiving the combined treatment (combo) manifested substantial enhancements in ovarian morphology, with a more conspicuous improvement effect than that achieved by astaxanthin or CUR alone (Figure 2).

### 2.3 The Synergistic Use of Astaxanthin and CUR Significantly Reduces Serum TC and LDL‐C Levels in PCOS Mice

**FIGURE 3:**
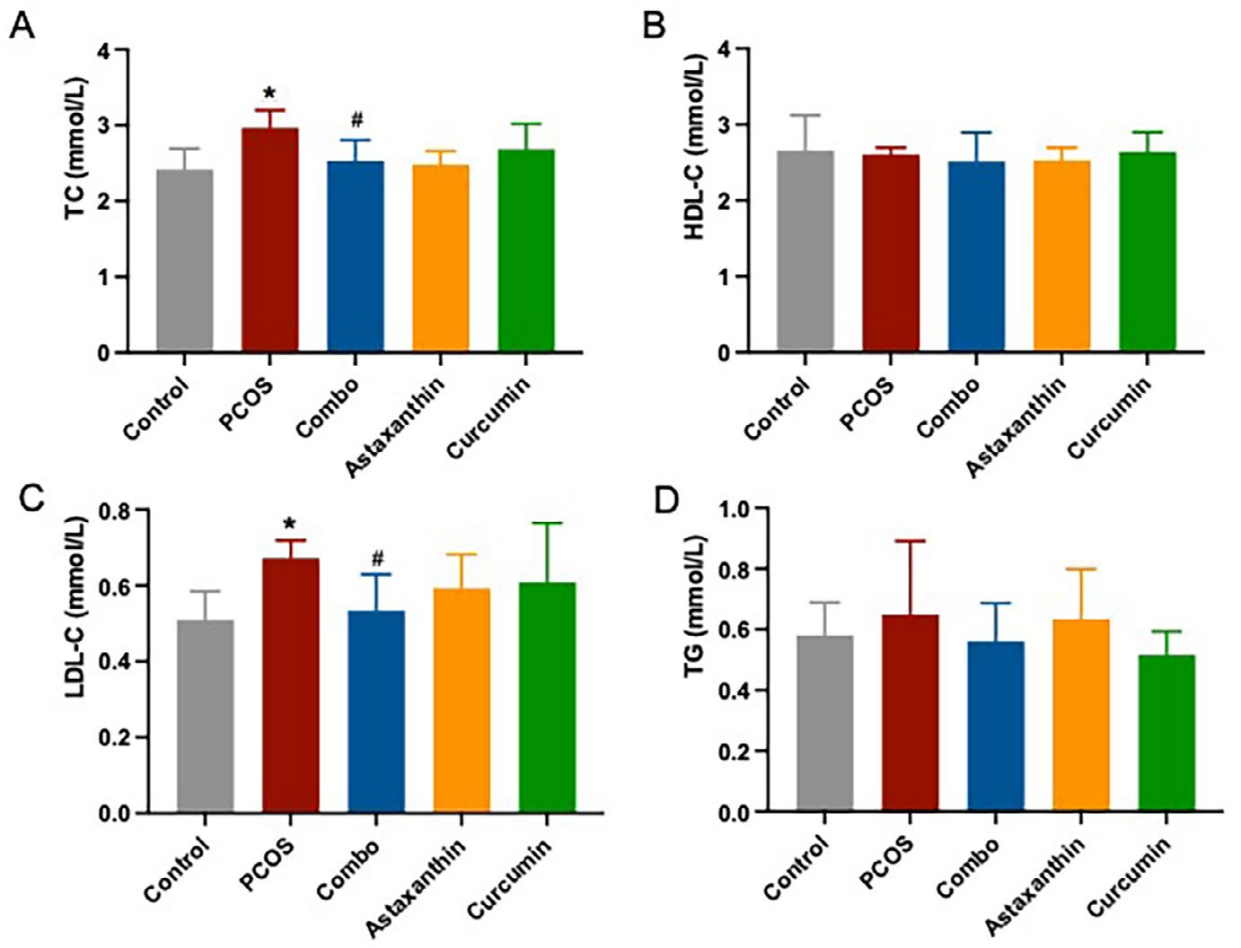
TC, HDL‐C, LDL‐C, and TG levels in serum. (A) Serum TC level; (B) serum HDL‐C level; (C) serum LDL‐C level; (D) serum TG level. *: versus control; #: versus PCOS. *p < 0.05, ^#^ *p* < 0.05. C, total cholesterol; Combo, astaxanthin combined with curcumin; HDL‐C, high‐density lipoprotein cholesterol; LDL‐C, low‐density lipoprotein cholesterol; PCOS, polycystic ovary syndrome; TG, triglyceride.

Notably, the PCOS mice presented with significantly elevated serum levels of TC and LDL‐C compared to the normal controls (Figure 3A,C, *p* < 0.05). In contrast to the PCOS group, the combined treatment group exhibited a significant decline in serum TC and LDL‐C concentration (Figure 3A,C, *p* < 0.05). However, there were no significant differences in HDL‐C and TG levels across the groups (Figure 3B,D, *p* > 0.05).

### 2.4 The Synergistic Use of Astaxanthin and CUR Significantly Improve Reproductive Endocrine Hormone Levels in PCOS Mice

**FIGURE 4:**
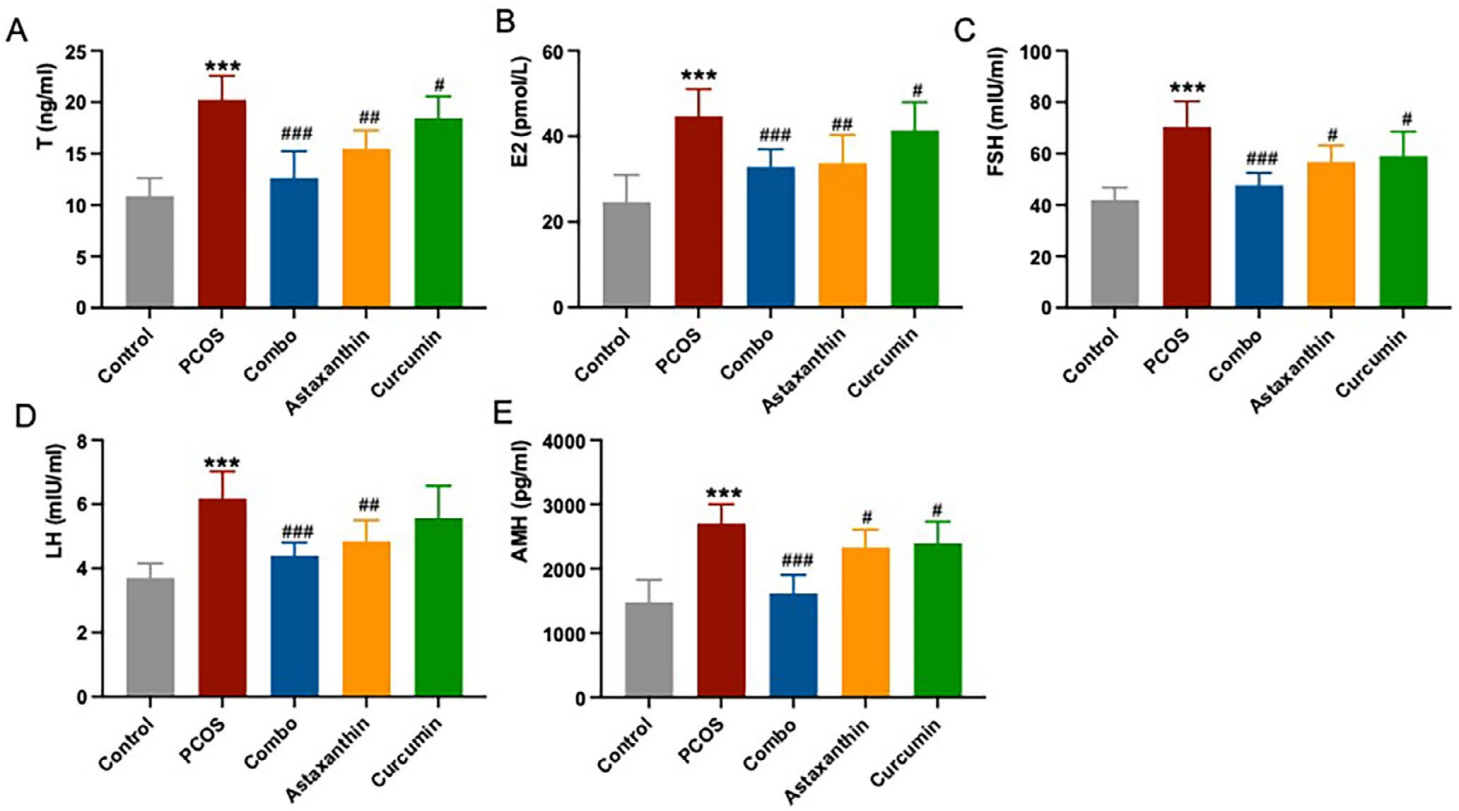
Reproductive hormones (T, E2, FSH, LH, and AMH) in serum. (A) Serum T level; (B) serum E2 level; (C) serum FSH level; (D) serum LH level; (E) serum AMH level. *: versus control; #: versus PCOS. ***p < 0.001, ^#^ *p* < 0.05, ^##^ *p* < 0.01, ^###^ *p* < 0.001. AMH, anti‐Müllerian hormone; Combo, astaxanthin combined with curcumin; E2, estradiol; FSH, follicle‐stimulating hormone; LH, luteinizing hormone; PCOS, polycystic ovary syndrome; T, testosterone.

The serum levels of T, E2, FSH, LH, and AMH were found to be significantly elevated in PCOS mice relative to the normal controls (Figure 4A–E, *p* < 0.001). Remarkably, when compared to the PCOS group, the combined treatment group displayed a significant reduction in serum levels of T, E2, FSH, LH, and AMH, with these reductions being more pronounced than those observed in mice treated with astaxanthin or CUR alone (Figure 4A–E, *p* < 0.001).

These results indicate that the synergistic use of astaxanthin and CUR significantly improves reproductive endocrine hormone levels in PCOS mice.

### 2.5 The Synergistic Use of Astaxanthin and CUR Significantly Improves the Systemic Oxidative Stress in PCOS Mice

**FIGURE 5:**
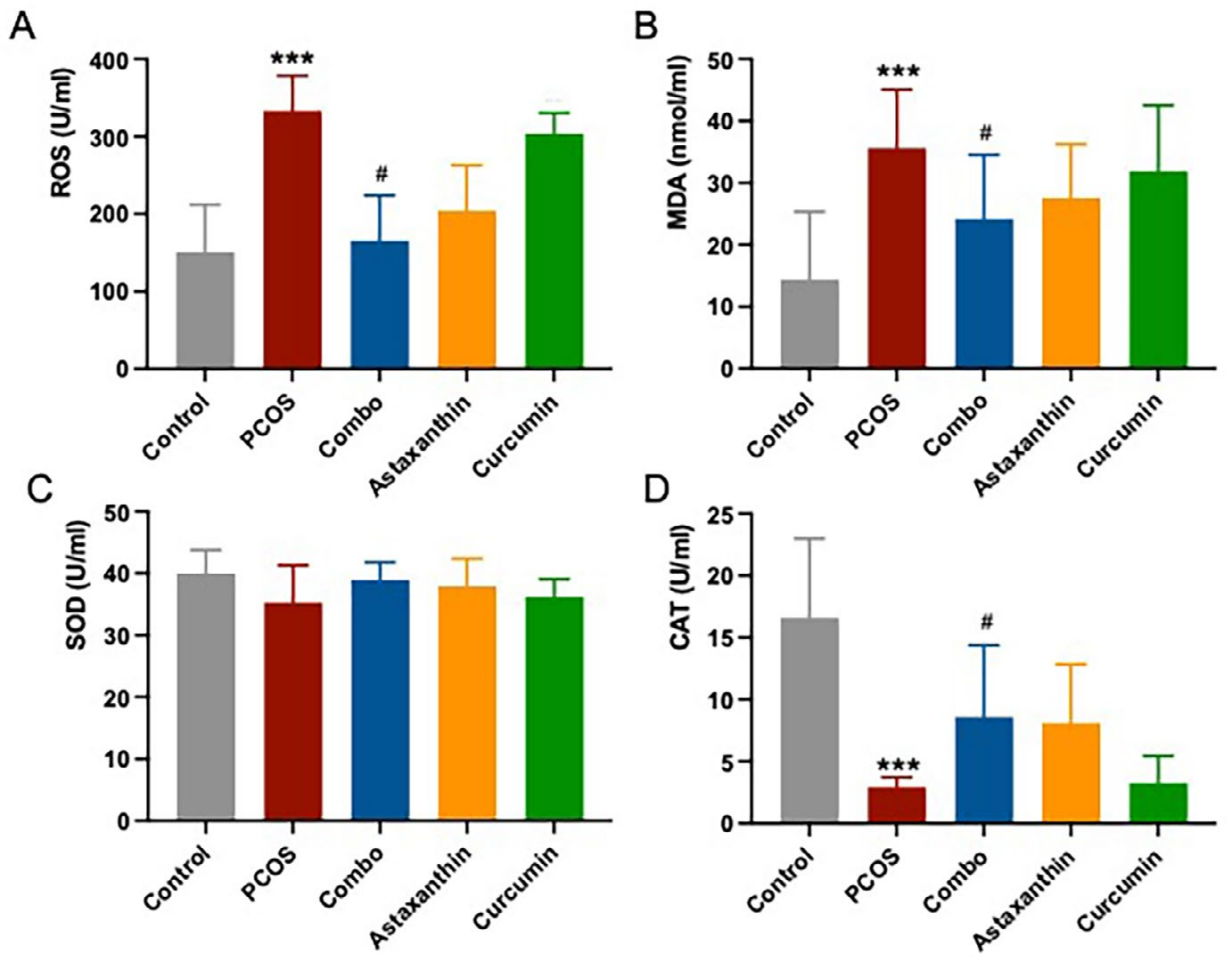
Serum oxidative stress (ROS, MDA, SOD, and CAT) levels. (A) serum ROS level; (B) serum MDA level; (C) serum SOD level; (D) serum CAT level. *: versus control; #: versus PCOS. ***p < 0.001, ^#^ *p* < 0.05. CAT, catalase; Combo, astaxanthin combined with curcumin; MDA, malondialdehyde; PCOS, polycystic ovary syndrome; ROS, reactive oxygen species; SOD, superoxide dismutase.

Serum levels of ROS and MDA were significantly higher in PCOS mice compared to normal controls (Figure 5A,B, *p* < 0.001), whereas CAT levels were significantly lower (Figure 5D, *p* < 0.001). In contrast, the mice in the combined treatment group exhibited significantly reduced ROS and MDA levels and significantly increased CAT levels in their serum compared to the PCOS group (Figure 5A,B,D, *p* < 0.05), demonstrating a more effective improvement over astaxanthin or CUR alone. No significant differences in SOD levels were observed across the groups (Figure 5C, *p* > 0.05).

### 2.6 The Synergistic Use of Astaxanthin and CUR Significantly Improves Oxidative Stress in Ovarian Tissue of PCOS Mice

**FIGURE 6:**
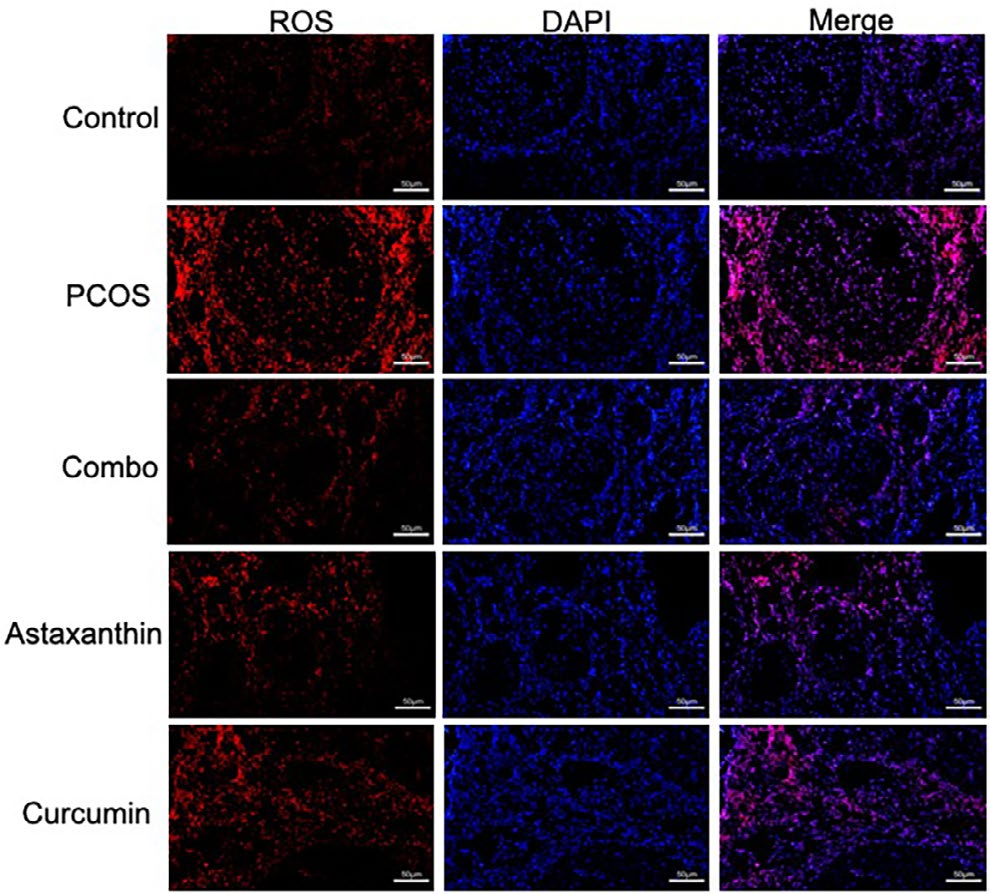
Ovarian ROS staining. Combo, astaxanthin combined with curcumin; PCOS, polycystic ovary syndrome; ROS, reactive oxygen species.

The results from ROS staining showed that the ovarian tissue in PCOS mice exhibited higher ROS levels compared to the control group. In contrast, the combined group demonstrated a significant decrease in ROS levels within the ovaries compared to the PCOS group; this improvement was notably more pronounced than that seen in the astaxanthin or CUR groups alone (Figure 6).

**FIGURE 7:**
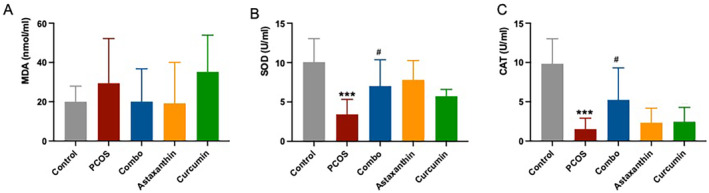
Ovarian oxidative stress (MDA, SOD, and CAT) levels. (A) serum MDA level; (B) serum SOD level; (C) serum CAT level. *: versus control; #: versus PCOS. ***p < 0.001, ^#^ *p* < 0.05. CAT, catalase; Combo, astaxanthin combined with curcumin; MDA, malondialdehyde; PCOS, polycystic ovary syndrome; SOD, superoxide dismutase.

The results showed that compared with the normal control group, the ovarian levels of SOD and CAT in the PCOS group were significantly decreased (Figure 7B,C, *p* < 0.001). However, in the combined group, both SOD and CAT levels were significantly elevated relative to the PCOS group (Figure 7B,C, *p* < 0.05), indicating a more pronounced improvement effect over either astaxanthin or CUR alone. No significant difference in MDA was found among all the groups (Figure 7A, *p* > 0.05).

These results indicate that the synergistic combination of astaxanthin and CUR can effectively mitigate oxidative stress state in the PCOS ovary to a certain extent.

### 2.7 The Synergistic Use of Astaxanthin and CUR Significantly Improves Chronic Ovarian Inflammatory Injury in PCOS Mice

**FIGURE 8:**
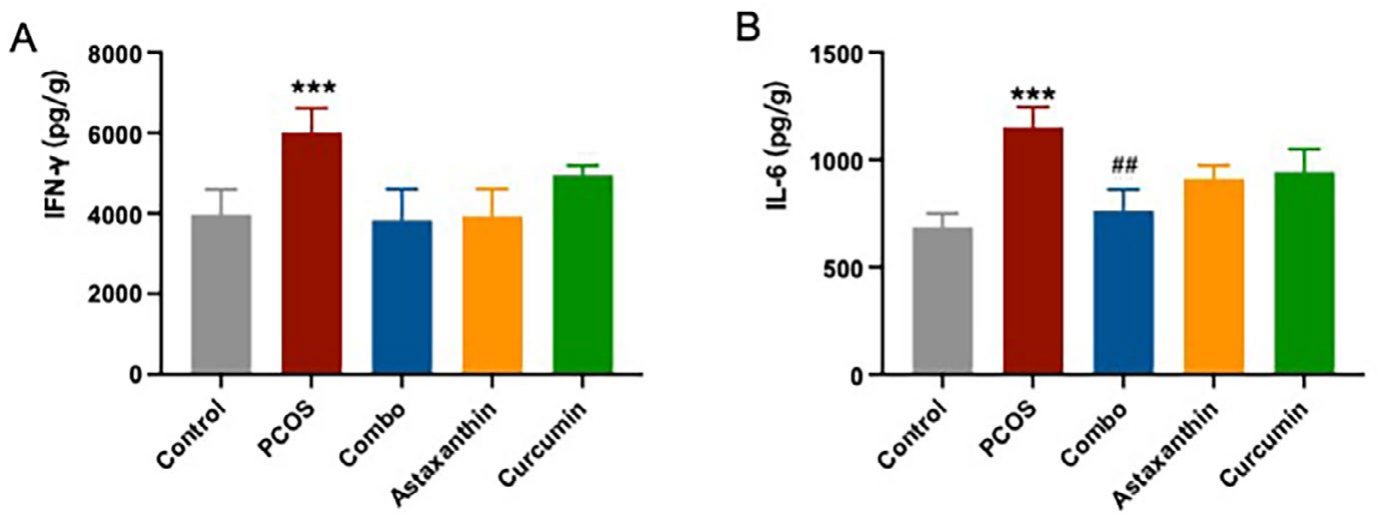
Ovarian inflammatory marker (IFN‐γ and IL‐6) levels. (A) IFN‐γ level; (B) IL‐6 level. *: versus control; #: versus PCOS. ***p < 0.001, ^##^ *p* < 0.01. Combo, astaxanthin combined with curcumin; IFN‐γ, interferon‐γ; IL‐6, interleukin‐6; PCOS, polycystic ovary syndrome.

The levels of IFN‐γ and IL‐6 in ovarian tissues from PCOS mice were significantly elevated compared to those in the normal control group (Figure 8A,B, *p* < 0.001). In contrast, the combination group had a significant reduction in IL‐6 levels within the ovarian tissues as compared to the PCOS group; this improvement was notably more obvious than that of either astaxanthin or CUR group alone (Figure 8A,B, *p* < 0.01).

These results indicate that the synergistic combination of astaxanthin and CUR can significantly improve chronic ovarian inflammation in PCOS mice, and this improvement effect is better than that of astaxanthin or CUR alone.

### 2.8 The Synergistic Use of Astaxanthin and CUR Significantly Improves the Number and Quality of Ovulation in PCOS Mice

**FIGURE 9:**
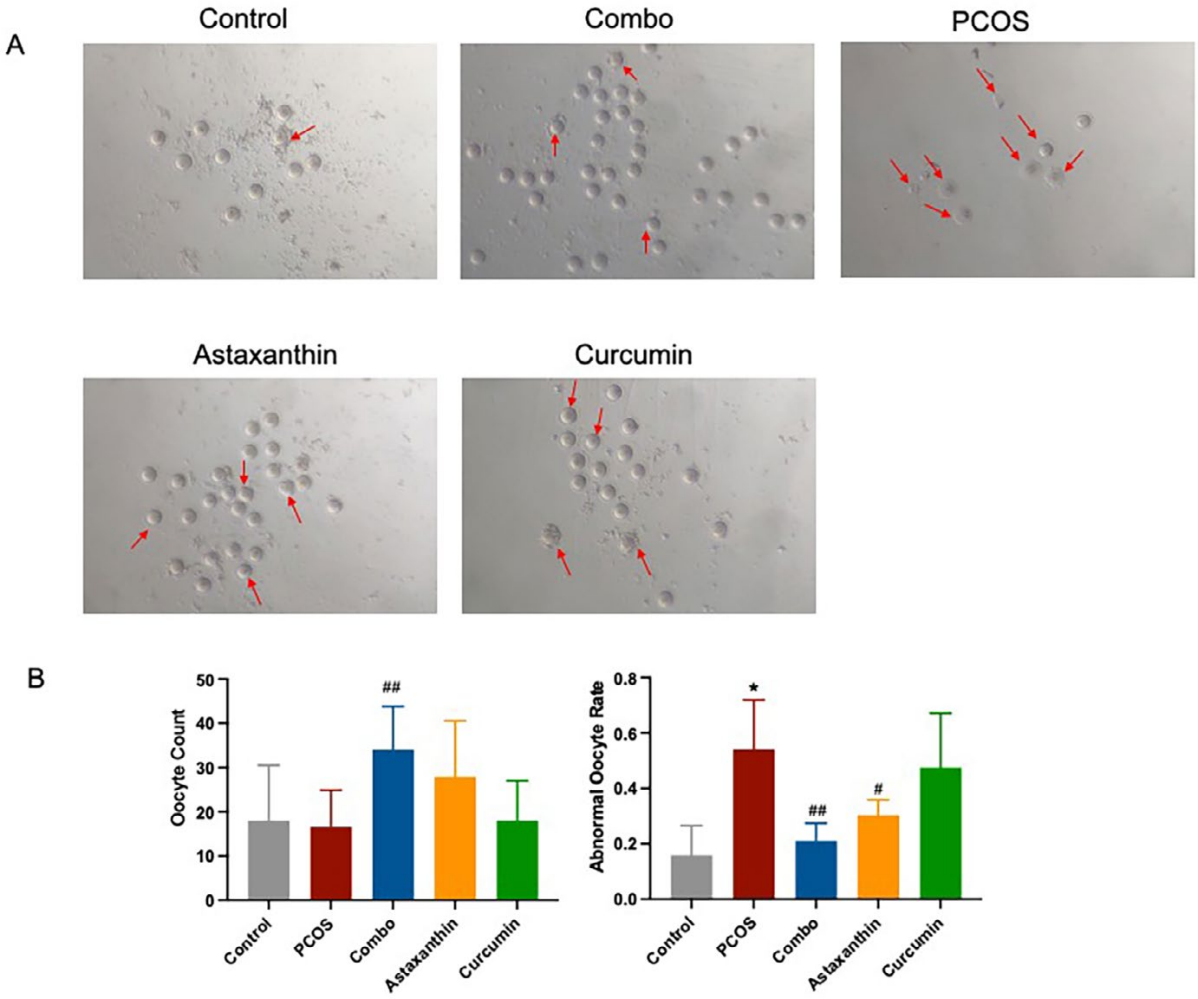
The number and quality of mouse ovulation. (A) The number and quality of ovulation. The arrow indicates an abnormal ovum; (B) statistical analysis of ovum quantity and quality. *: versus control; #: versus PCOS. *p < 0.05, ^##^ *p* < 0.05, ^##^ *p* < 0.01. Combo, astaxanthin combined with curcumin; PCOS, polycystic ovary syndrome.

Compared to the normal control group, the PCOS mice had a significantly reduced number of oocytes accompanied by a significantly higher incidence of oocytes with abnormal morphologies (Figure 9A,B, *p* < 0.001). In comparison with the PCOS group, the combined group experiences a significant increase in the quantity of ova, along with a notable decrease in the percentage of ova displaying abnormalities (Figure 9A,B, *p* < 0.05). This improvement effect was more obvious than that of astaxanthin or CUR alone.

These results indicate that the synergistic use of astaxanthin and CUR significantly improves the quantity and quality of ovulation in PCOS mice, and the improvement effect is better than that of astaxanthin or CUR alone.

## 3 Discussion

In this study, PCOS mice were treated with astaxanthin, CUR, or a combination of both, aiming to explore the potential improvement of ovarian function and its mechanism in PCOS mice. The results showed that the synergistic use of astaxanthin and CUR exerted a significantly positive influence on lipid metabolism and reproductive endocrine function in PCOS mice. Moreover, it effectively alleviated oxidative stress and chronic inflammatory injury in both systemic and ovarian tissues, thereby promoting follicular development and ovulation in PCOS mice.

The pathogenesis of PCOS is multifaceted, involving genetic polymorphism, genetic factors, and metabolic syndromes, among others. This condition not only impacts female reproductive function but still can heighten the risk of glucose and lipid metabolic disorders. Despite numerous studies on PCOS, its complete etiology and pathological mechanisms remain unclear, and several challenges persist in its clinical management. Identifying food‐grade agents with high safety profiles for PCOS treatment holds immense significance in addressing this health issue, thus presenting a promising avenue for future therapeutic strategies.

In recent years, the role of astaxanthin in the reproductive system has attracted more and more attention. Studies have demonstrated that astaxanthin effectively mitigates endoplasmic reticulum stress and insulin resistance in PCOS patients (Jabarpour et al. 2023, 2024). In PCOS patients' granulosa cells, astaxanthin treatment has been shown to increase both plasma total antioxidant capacity (TAC) and activation of the nuclear factor erythroid 2‐related factor 2 (Nrf2) axis (Gharaei et al. 2022). A randomized clinical trial showed AST pretreatment may modify inflammation and improve assisted reproductive technology (ART) outcomes in PCOS infertile patients (Fereidouni et al. 2024). In addition to that, supplementation of 0.5 mg/kg of natural astaxanthin to in vitro cultures of porcine oocytes significantly improves their maturation, fertilization, and development under heat‐stress conditions (Do et al. 2015). Moreover, when applied to in vitro–cultivated bovine luteal cells, astaxanthin can increase progesterone synthesis (Kamada et al. 2017). In zebrafish, astaxanthin can alleviate oxidative stress and apoptosis caused by some chemical stimuli, thereby enhancing embryonic growth (Zhang et al. 2024). Clinical studies have shown that astaxanthin has a certain effect on improving the oocyte quality of PCOS patients undergoing in vitro fertilization (IVF) (Gharaei et al. 2022), suggesting a potentially beneficial impact on ovarian function.

CUR has a variety of pharmacological effects including anti‐inflammatory, antitumor, antiatherosclerotic, antioxidant, and regulation of lipid metabolism. Research has indicated that CUR contributes to relieving ovarian dysfunction and offers a protective effect on ovarian health (Yan et al. 2018). Tiwari‐pandey et al. showed that CUR could promote the proliferation of mouse ovarian cells, promote oocyte production and follicular development, and inhibit apoptosis in follicular cells (Tiwari‐Pandey and Sairam 2009). A randomized double‐blind placebo‐controlled clinical trial showed CUR could reduce fasting plasma glucose (FPG) and dehydroepiandrosterone levels in individuals with PCOS (Heshmati et al. 2021). Additionally, studies have shown that CUR exhibits protective effects on ovarian structures and folliculogenesis in the diabetic rat ovary (Tufekci and Kaplan 2023). In PCOS rats, CUR treatment exerted protective effects on the ovaries, downregulated serum testosterone, restored IR, inhibited inflammatory cell infiltration in ovarian tissues, decreased IRS1, PI3K, and AKT expressions, and upregulated GLUT4 and PTEN expressions (Zheng et al. 2022). Recent research suggested that CUR is therapeutically effective for PCOS and is comparable to metformin (Shannag et al. 2024).

Although previous research points to the individual benefits of astaxanthin or CUR on ovarian function, the synergistic effects of astaxanthin and CUR have not been reported, nor has it been established whether their combination yields superior efficacy. In this study, we found that the combination of astaxanthin and CUR significantly improves estrous cycle irregularities, lipid metabolism, reproductive endocrine parameters, oxidative stress, and inflammation levels, and finally, improves the quantity and quality of oocytes in PCOS mice.

In conclusion, the synergistic effects of astaxanthin and CUR significantly improve lipid metabolism and reproductive endocrine function in PCOS mice. It effectively reduces oxidative stress and chronic inflammatory damage within systemic and ovarian tissues, and it promotes follicular growth and ovulation in PCOS mice. These findings underscore the potential value of combining astaxanthin and CUR for treating PCOS‐related complications.

## Author Contributions

**Gao Zhang:** project administration (equal), writing – original draft (equal). **Man He:** project administration (equal), writing – original draft (equal). **Zun Wang:** methodology (equal). **Jinrong Zheng:** software (equal). **Dan Zhao:** methodology (equal). **Li Nie:** investigation (equal). **Dongzhi Yuan:** investigation (equal). **Ziyu Zhao:** methodology (equal). **Wei Wang:** software (equal). **Limin Yue:** formal analysis (equal), funding acquisition (equal), supervision (equal), writing – review and editing (equal).

## Conflicts of Interest

The authors declare no conflicts of interest.

## Data Availability

Research data are not shared.
